# Advantages of deep learning reconstruction algorithm in ultra-high-resolution CT for the diagnosis of pancreatic cystic neoplasm

**DOI:** 10.1007/s11604-025-01804-7

**Published:** 2025-05-30

**Authors:** Keitaro Sofue, Yoshiko Ueno, Shinji Yabe, Eisuke Ueshima, Takeru Yamaguchi, Atsuhiro Masuda, Arata Sakai, Hirochika Toyama, Takumi Fukumoto, Masatoshi Hori, Takamichi Murakami

**Affiliations:** 1 Department of Radiology, Kobe University Graduate School of Medicine, 7-5-2, Kusunoki-Cho, Chuo-Ku, Kobe, 650-0017 Japan; 2 Division of Gastroenterology, Department of Internal Medicine, Kobe University Graduate School of Medicine, Kobe, Japan; 3 Division of Hepato-Biliary-Pancreatic Surgery, Department of Surgery, Kobe University Graduate School of Medicine, Kobe, Japan; 4 Department of Artificial Intelligence in Diagnostic Radiology, Osaka University Graduate School of Medicine, Osaka, Japan

**Keywords:** Computed tomography, Deep learning, Pancreas, Cystic lesion, Intraductal papillary mucinous neoplasm

## Abstract

**Purpose:**

This study aimed to evaluate the image quality and clinical utility of a deep learning reconstruction (DLR) algorithm in ultra-high-resolution computed tomography (UHR-CT) for the diagnosis of pancreatic cystic neoplasms (PCNs).

**Methods:**

This retrospective study included 45 patients with PCNs between March 2020 and February 2022. Contrast-enhanced UHR-CT images were obtained and reconstructed using DLR and hybrid iterative reconstruction (IR). Image noise and contrast-to-noise ratio (CNR) were measured. Two radiologists assessed the diagnostic performance of the imaging findings associated with PCNs using a 5-point Likert scale. The diagnostic performance metrics, including sensitivity, specificity, and area under the receiver operating characteristic curve (AUROC), were calculated. Quantitative and qualitative features were compared between CT with DLR and hybrid IR. Interobserver agreement for qualitative assessments was also analyzed.

**Results:**

DLR significantly reduced image noise and increased CNR compared to hybrid IR for all objects (*p* < 0.001). Radiologists rated DLR images as superior in overall quality, lesion delineation, and vessel conspicuity (*p* < 0.001). DLR produced higher AUROC values for diagnostic imaging findings (ductal communication: 0.887‒0.938 vs. 0.816‒0.827 and enhanced mural nodule: 0.843‒0.916 vs. 0.785‒0.801), although DLR did not directly improve sensitivity, specificity, and accuracy. Interobserver agreement for qualitative assessments was higher in CT with DLR (κ = 0.69‒0.82 vs. 0.57‒0.73).

**Conclusion:**

DLR improved image quality and diagnostic performance by effectively reducing image noise and improving lesion conspicuity in the diagnosis of PCNs on UHR-CT. The DLR demonstrated greater diagnostic confidence for the assessment of imaging findings associated with PCNs.

## Introduction

Over the past decades, advancements in imaging have significantly improved the detection and characterization of pancreatic cystic lesions [1]. Pancreatic cysts are pathologically classified as non-neoplastic or pancreatic cystic neoplasms (PCNs), including intraductal papillary mucinous neoplasms (IPMNs), mucinous cystic neoplasms, serous cystic neoplasms, and solid pseudopapillary neoplasm [2]. Given the wide spectrum of malignant potential associated with PCNs, accurate diagnosis and risk stratification are essential for optimal management and follow-up strategies [3, 4]

PCNs are typically evaluated using computed tomography (CT), magnetic resonance imaging (MRI), and endoscopic ultrasound (EUS). The diagnostic criteria include cyst size, morphology, septa thickness, enhancing mural nodules, and communication with the main pancreatic duct (MPD). Contrast-enhanced EUS (CE-EUS) provides detailed visualization of intra-cystic structures and microcirculation owing to its high spatial and contrast resolution [5–7]. However, CE-EUS has drawbacks, including its invasiveness, operator dependency, and restricted use for high-risk cases [3, 4]. Therefore, accurate CT and MRI evaluation remains crucial for identifying patients who require further examination using CE-EUS.

Recently, ultra-high-resolution CT (UHR-CT), featuring smaller detector elements and tube focus sizes than conventional CT, has become clinically available. UHR-CT achieves higher spatial resolution and has proven clinical utility in the lungs, coronary arteries, temporal bone, and peripheral arteries [8–11]. For PCN diagnosis, the higher spatial resolution of UHR-CT could potentially improve lesion characterization. However, UHR-CT also increases image noise owing to the smaller detector elements, posing a challenge for clinical use, particularly in abdominal imaging [12, 13].

To mitigate this issue, a new deep learning reconstruction (DLR) algorithm (Advanced intelligent Clear-IQ Engine, AiCE Body Sharp, Canon Medical Systems) was introduced. DLR is based on deep convolutional neural networks trained with high- and low-quality image pairs to effectively distinguish true signals from noise and artifacts [14, 15]. This algorithm effectively removes low-frequency noise while preserving fine structural details, which contributes to improved spatial resolution and image texture. Compared with conventional hybrid iterative reconstruction (IR) algorithms (Adaptive Iterative Dose 3 Dimensional, AIDR 3D; Canon Medical Systems), the DLR algorithm has demonstrated improved image quality on UHR-CT [16–18]. However, the clinical utility of UHR-CT with DLR for diagnosing PCNs has not been fully elucidated.

This study aimed to evaluate the image quality and clinical utility of the DLR algorithm in UHR-CT for diagnosing PCNs.

## Materials and methods

### Study population

This retrospective study was approved by the institutional review board, with a waiver for written informed consent. Between March 2020 and February 2022, 219 consecutive patients underwent CE-CT and UHR-CT to screen pancreatic diseases. The inclusion criteria were as follows: (a) patients with PCNs who received curative or conservative treatment, and (b) patients who underwent CE-EUS as the reference standard. The exclusion criteria were as follows: (a) intervals of CE-CT and CE-EUS of more than 3 months for patients who underwent surgical resection for PCNs, and (b) CE-EUS was not performed before or after CE-CT for patients who did not undergo surgery. We included only cases in which CE-EUS was performed both before and after the UHR-CT for patients who did not undergo surgery, to confirm that there were no interval changes in the PCNs.

**Fig. 1:**
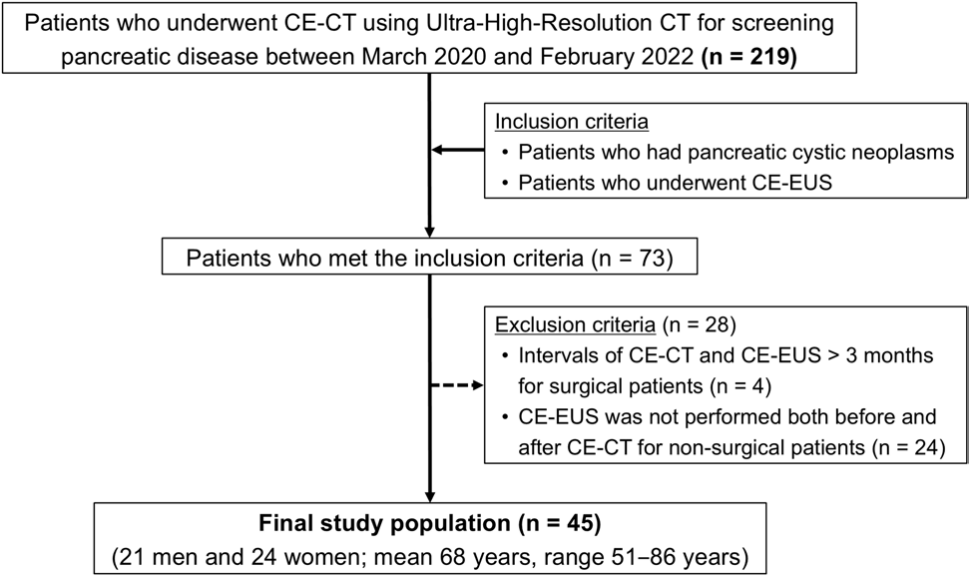
Flowchart of the study population. *CE-CT* Contrast-enhanced computed tomography, *CE-EUS* Contrast-enhanced endoscopic ultrasound

Seventy-three patients met the inclusion criteria for this analysis. Of these, 28 patients were excluded because the intervals of CE-CT and CE-EUS were more than 3 months and CE-EUS was not performed before or after CE-CT. Consequently, the final study population consisted of 45 patients (21 men and 24 women; mean age, 68 ± 8 years; range, 51–86 years). The patient enrollment process is shown in Fig. 1, and the patient characteristics are shown in Table 1.

**Table 1:** Patient demographics, tumor characteristics, and CE-EUS findings

Characteristics	Total (*n* = 45)
Patient demographics	
Age (years)	67.7 ± 8.4 (51‒86)
Sex	
Male	21 (46.7)
Female	24 (53.3)
Body mass index	22.1 ± 2.6 (15.4‒29.4)
Radiation exposure	
Pancreatic phase	
CTDI_vol_ (mGy)	17.2 ± 4.7 (9.8‒26.8)
DLP (mGy × cm)	471.9 ± 161.5 (247.4‒1003.3)
Portal venous phase	
CTDI_vol_ (mGy)	17.2 ± 4.7 (9.8‒26.8)
DLP (mGy × cm)	472.7 ± 162.8 (246.8‒1011.2)
Tumor characteristics	
Number of lesions per patient	
1	27 (60)
2	9 (20)
≥ 3	9 (20)
The largest lesion size (mm)	24.4 ± 13.0 (5.2‒60.4)
Location of lesion	
Head	17 (37.8)
Body/tail	21 (46.7)
Diffuse	7 (15.6)
CE-EUS findings	
Continuity between the cyst and pancreatic duct	
Present	38 (84.4)
Absent	7 (15.6)
Enhanced thickened wall or enhanced mural nodule	
≥ 5 mm	8 (17.8)
< 5 mm	7 (15.6)
Unenhanced mural nodule	5 (11.1)
Absent	25 (55.6)

Unless otherwise specified, categorical data are numbers of patients, with percentages in parentheses. Continuous data are means ± standard deviations, with ranges in parentheses

*CTDI*_*vol*_ volume CT dose index, *DLP* dose-length product, *CE-EUS* contrast-enhanced endoscopic ultrasound

### CT technique

All CE-CT images were acquired using a UHR-CT scanner (Aquilion Precision, Canon Medical Systems). After obtaining pre-contrast images, an iodinated nonionic contrast agent (600 mg/kg) was intravenously injected using a power injector for a fixed duration of 30 s. Pancreatic phase (PP), portal venous phase (PVP), and equilibrium phase images were obtained 37‒45, 70, and 180 s after contrast injection. The scanning delay for PP images was determined using the bolus-tracking technique with a delay of 20 s after a trigger threshold (80 HU) in the abdominal aorta at the level of the first lumbar vertebral body. PP and PVP images were obtained in super-high-resolution mode with the following scanning parameters: 1792 channels per detector row, 160 × 0.25 mm; matrix size, 1024; rotation time, 0.75 s; pitch factor, 0.806; tube voltage, 120 kVp; and tube current regulated by automatic exposure control with a standard deviation of 18.

For quantitative and qualitative analyses, two separate image sets were reconstructed from the same raw data in PP and PVP images: hybrid IR (AIDR 3D, FC13, mild) and DLR (AiCE Body Sharp, standard) both in 1024 × 1024 matrix at 0.25 mm slice thickness. The reconstruction strengths were selected based on our institution’s standard clinical protocol for abdominal imaging. Although pre-contrast and equilibrium phase images were obtained as part of a clinical protocol, they were not evaluated in this study. The CT dose index (CTDl_vol_) and dose-length product (DLP) were recorded for radiation exposure assessment.

### Quantitative image analysis

**Fig. 2:**
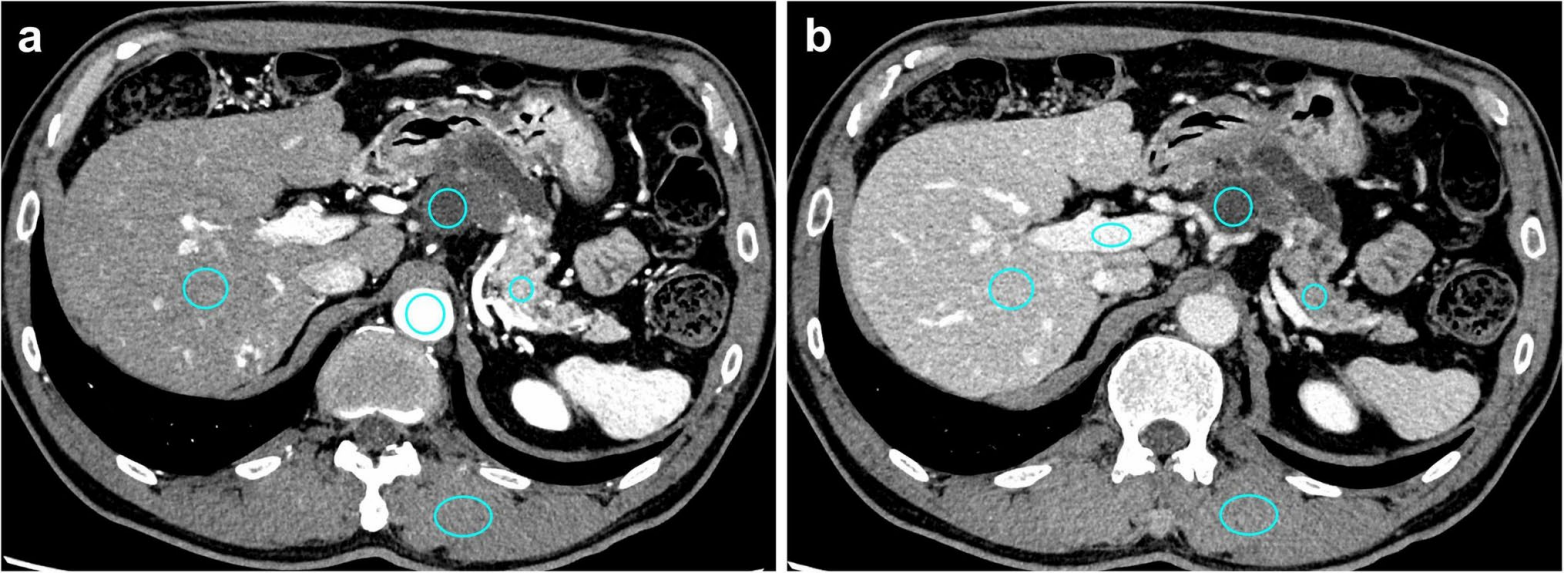
UHR**-**CT images reconstructed with DLR on **a** pancreatic phase and **b** portal venous phase images in a 56-year-old man with pathologically confirmed invasive adenocarcinoma derived from IPMN. For CT attenuation measurements, regions of interest were manually drawn on the pancreatic cystic neoplasm, pancreatic parenchyma, liver parenchyma, abdominal aorta (pancreatic phase only), portal vein (portal venous phase only), and paraspinal muscle. *UHR-CT* Ultra-high-resolution computed tomography, *DLR* Deep learning reconstruction, *IPMN* Intraductal papillary mucinous neoplasm

Quantitative measurements were performed by an abdominal radiologist (E.U. with 15 years of experience in abdominal imaging) on a dedicated workstation (Vitrea version 1.0; Canon Medical Systems). For CT attenuation measurements (i.e., Hounsfield units [HUs]), regions of interest (ROIs) were manually placed within the PCNs, pancreatic parenchyma, liver parenchyma, abdominal aorta (PP images alone), portal vein (PVP images alone), and paraspinal muscles. The ROIs were carefully placed in homogeneous areas to avoid predominant artifacts, large vessels, calcification, and macroscopic fat infiltration. The ROIs of the PCNs were placed on the cystic component without an enhanced nodule of the largest lesion (Fig. 2). For all measurements, the size, shape, and position of the ROIs were maintained between the hybrid IR and DLR reconstruction algorithms by applying a copy-and-paste function on the workstation. To achieve data consistency, all quantitative measurements for each patient and each object were performed twice, and the averaged values were used for further analyses.

For each image set, the contrast-to-noise ratio (CNR) of the objects and pancreas-to-lesion CNR were calculated using the following formulas: − 

$$CNR_{object} = \, \left( {HU_{object} \, - \, HU_{paraspinal \, muscle} } \right) \, / \, SD_{noise}$$

$$CNR_{pancreas - to - lesion} = \, \left( {HU_{pancreas} - \, HU_{lesion} } \right) \, / \, SD_{noise}$$

where HU_object_, HU_paraspinal muscle_, and HU_pancreas_ are the attenuations of the object, the paraspinal muscle, and the pancreas, respectively. SD_noise_ is the standard deviation of the paraspinal muscle. We used CT values and SD of the paraspinal muscle as the indicator of reference and image noise for the objects based on previous papers [16, 19, 20].

### Qualitative image analysis

Two board-certified abdominal radiologists (S.Y. and K.S., with 6 and 19 years of experience in abdominal imaging, respectively) independently reviewed the two imaging datasets reconstructed with hybrid IR and DLR using the same workstation (Vitrea, version 1.0). The imaging datasets were reviewed in a random order, and the readers were blinded to any clinical information, except that the patients had PCNs. The preset window width and level were 280 and 60 HU, respectively, and the readers were allowed to change these settings as appropriate.

Subjective image quality assessments were performed on both the PP and PVP images. The readers assessed the overall image quality, image noise, sharpness, vessel conspicuity, and lesion delineation rated on a 5-point scale: 1 = unacceptable, 2 = suboptimal, 3 = acceptable, 4 = good, and 5 = excellent. The readers also assessed the presence or absence of the following imaging findings: (a) continuity between the cystic lesion and the MPD and (b) enhanced thickened wall or enhanced mural nodule. These assessments were based on the 2017 international consensus guidelines for the management of pancreatic IPMN and MCN, which use such findings to subjectively differentiate malignant from benign lesions [3]. This assessment was conducted using a 5-point confidence scale and rated as follows: 1 = absent; 2 = probably absent; 3 = indeterminate; 4 = probably present; and 5 = present. The evaluation was based on axial PP and PVP images. In cases of disagreement between the two readers, the final decision was made through a consensus review after the reading session. CE-EUS examinations were used as the reference standards for these findings.

### Statistical analysis

Continuous variables were calculated as mean ± standard deviation, while categorical variables were presented as counts and frequencies. Wilcoxon signed-rank tests were used to evaluate the differences in image noise, CNRs, and subjective image quality assessments between the reconstructed images from hybrid IR and DLR. For qualitative image analysis, interobserver agreement was also evaluated using weighted kappa statistics, the values of which were interpreted as follows: 0‒0.20 (poor), 0.21‒0.40 (fair), 0.41‒0.60 (moderate), 0.61‒0.80 (substantial), and 0.81‒1.00 (excellent).

To assess the subjective diagnosis of the image findings, receiver operating characteristic (ROC) analysis was performed to evaluate the diagnostic performance of the two imaging datasets for each reader. The areas under the ROC curves (AUROCs) were estimated non-parametrically and compared using the DeLong test between the two imaging datasets. The sensitivity, specificity, and accuracy were compared using McNemar’s test. A confidence scale of 4 or 5 indicated a positive diagnosis for each imaging finding.

A two-tailed *p* value < 0.05 was considered to indicate a significant difference. All statistical analyses were performed using SPSS version 27.0 (IBM Corporation).

## Results

### Patient characteristics

Patient characteristics are summarized in Table 1. The mean largest lesion size of PCNs in each patient was 24.4 ± 13.0 mm (range, 5.2‒60.4 mm). Using CE-EUS, PCNs were diagnosed as intraductal papillary mucinous neoplasms (*n* = 38), mucinous cystic neoplasms (*n* = 6), or serous cystic neoplasm (*n* = 1). Enhanced mural nodules > 5 mm were detected in nine cases on CE-EUS. Of these, surgery was performed in seven cases, revealing invasive ductal adenocarcinoma derived from IPMN (*n* = 5), pancreatic ductal adenocarcinoma concomitant with IPMN (*n* = 1), IPMN with high-grade dysplasia (*n* = 1), and adenocarcinoma from mucinous cystic neoplasm (*n* = 1). Pathological proof was not obtained in two cases due to surgical contraindications. Enhanced thickened walls or enhanced mural nodules < 5 mm were observed in four cases using CE-EUS. Of these, surgery was performed in two cases, and the pathological diagnoses were invasive ductal adenocarcinoma derived from IPMN (*n* = 1) and IPMN with low-grade dysplasia (*n* = 1), while the other two cases remained under observation.

The mean CTDI_vol_ and DLP values were 17.2 ± 4.7 mGy (range 9.8‒26.8) and 471.9 ± 161.5 mGy cm (range 247.4‒1003.3) in the PP images and 17.2 ± 4.7 mGy (range 9.8‒26.8) and 472.7 ± 162.8 mGy cm (range 246.8‒1011.2) in the PVP images, respectively.

### Quantitative image analysis

**Fig. 3:**
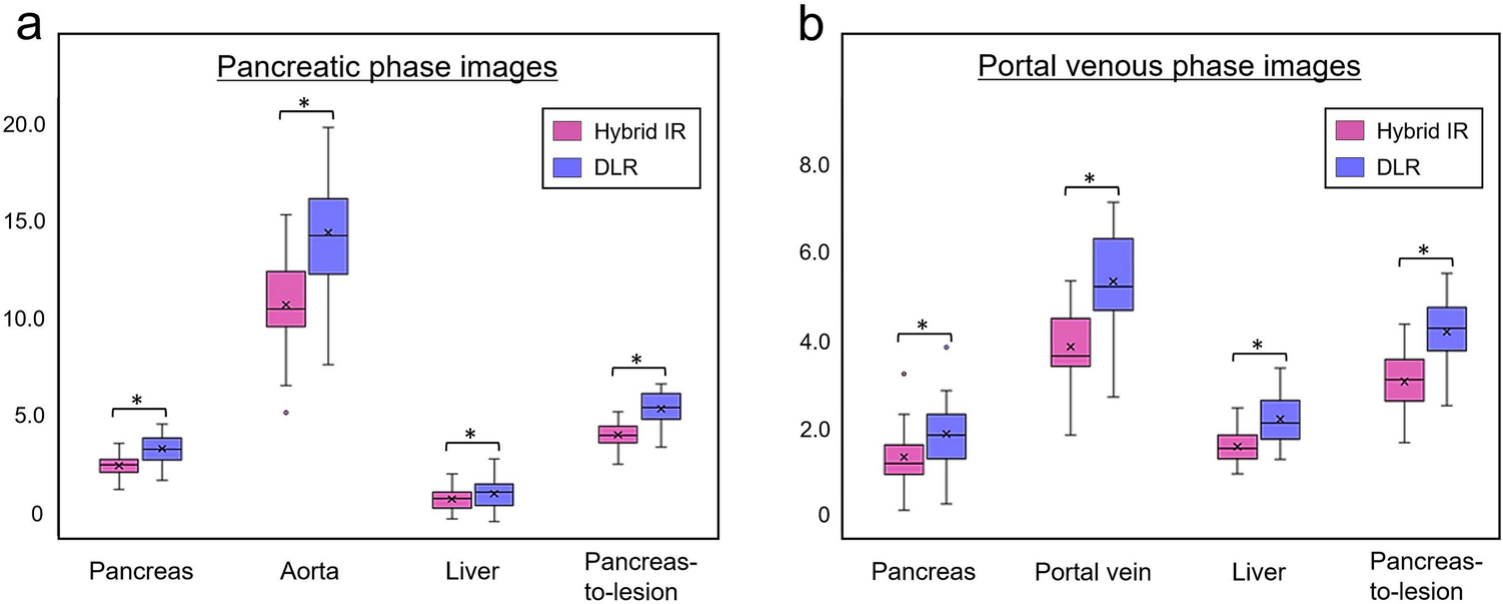
Contrast-to-noise ratio measurements of the pancreas, aorta, portal vein, liver, and pancreas-to-lesion in UHR-CT images reconstructed with hybrid IR and DLR on **a** pancreatic phase and **b** portal venous phase images. Error bars represent the standard deviations of the means obtained from 45 patients. *UHR-CT*, ultra-high-resolution computed tomography; *IR*, iterative reconstruction; *DLR*, deep learning reconstruction. * = significant difference; *P* < 0.001

Table 2 summarizes the details of the quantitative image analysis. Mean image noise was significantly lower on the images with DLR than those with hybrid IR both on PP (20.3 ± 1.9 vs. 27.8 ± 2.4, *p* < 0.001) and PVP (20.4 ± 1.6 vs. 28.2 ± 2.2, *p* < 0.001) images. While the CT values for each organ and PCNs on the images with DLR were slightly higher than those with hybrid IR, significant differences were observed in the pancreas, aorta, liver, and PCN on PP (*p* = < 0.001‒0.028) and in the pancreas, liver, and PCN on PVP (*p* = < 0.001‒0.003), respectively. The CNRs of the pancreas, aorta, portal vein, and liver were significantly higher in images with DLR than in those with hybrid IR (*p* < 0.001 for all). The pancreas-to-lesion CNRs on the images with DLR were significantly greater than those with hybrid IR both on PP phase (5.9 ± 1.2 vs. 4.3 ± 0.8, *p* < 0.001) and PVP phase (4.5 ± 0.9 vs. 3.3 ± 0.7, *p* < 0.001) images (Fig. 3).

**Table 2:** Comparisons of quantitative image analyses

	Hybrid IR	DLR	*p* value
Pancreatic phase			
Image noise	27.78 ± 2.36	20.26 ± 1.93	< 0.001
CT value			
Pancreas	135.44 ± 15.94	136.47 ± 15.93	0.026
Aorta	364.50 ± 59.89	365.36 ± 59.67	< 0.001
Liver	85.26 ± 13.94	86.57 ± 13.72	0.028
PCN	17.25 ± 9.02	18.02 ± 9.55	< 0.001
Paraspinal muscle	62.41 ± 6.21	63.01 ± 6.24	0.144
Contrast-to-noise ratio			
Pancreas	2.66 ± 0.68	3.68 ± 0.99	< 0.001
Aorta	10.93 ± 2.31	14.92 ± 3.17	< 0.001
Liver	0.84 ± 0.57	1.19 ± 0.81	< 0.001
Pancreas-to-lesion	4.29 ± 0.79	5.87 ± 1.20	< 0.001
Portal venous phase			
Image noise	28.24 ± 2.23	20.36 ± 1.63	< 0.001
CT value			
Pancreas	108.26 ± 16.01	109.58 ± 15.06	0.003
Portal vein	178.26 ± 18.56	179.12 ± 20.42	0.095
Liver	114.12 ± 9.42	115.44 ± 9.32	< 0.001
PCN	16.60 ± 9.99	17.41 ± 10.29	< 0.001
Paraspinal muscle	67.97 ± 6.65	69.00 ± 6.64	0.078
Contrast-to-noise ratio			
Pancreas	1.43 ± 0.59	2.00 ± 0.77	< 0.001
Portal vein	3.94 ± 0.72	5.44 ± 1.12	< 0.001
Liver	1.66 ± 0.38	2.32 ± 0.54	< 0.001
Pancreas-to-lesion	3.27 ± 0.66	4.51 ± 0.90	< 0.001

Data are means ± standard deviations

*IR* iterative reconstruction, *DLR* deep learning reconstruction, *PCN* pancreatic cystic neoplasm

### Qualitative image analysis

**Fig. 4:**
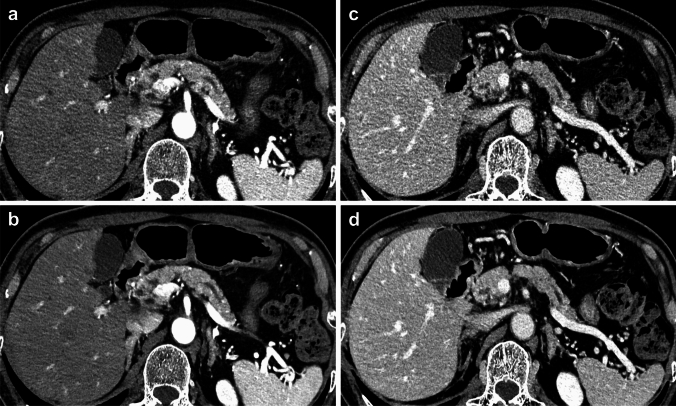
Abdominal UHR-CT on **a, b** pancreatic phase and **c, d** portal venous phase images in an 83-year-old man who had branch-duct type IPMN. Images were reconstructed with hybrid IR (upper low) and DLR (lower low). Image noise on the images with DLR were markedly lower than those with hybrid IR both on the pancreatic phase and portal venous phase images. Note that the improvement of image quality and better visualization not only for the abdominal vessels but also for abdominal organs and lesion delineation of the pancreas on the images with DLR were markedly lower than those with hybrid IR. *UHR-CT* Ultra-high-resolution computed tomography, *IPMN* Intraductal papillary mucinous neoplasm, *IR* Iterative reconstruction, *DLR* Deep learning reconstruction

For subjective image quality assessments, overall image quality was superior on the images with DLR (4.09 ± 0.68) compared to those with hybrid IR (2.98 ± 0.58) (*p* < 0.001) (Table 3). Especially in the image noise assessment, CT with DLR showed significant improvement (DLR: 3.92 ± 0.68 vs. hybrid IR: 2.61 ± 0.61) (*p* < 0.001). In addition, sharpness, vessel conspicuity, and lesion delineation were also significantly superior on CT with DLR than on CT with hybrid IR (*p* < 0.001 for all) (Fig. 4). Interobserver agreement between the two readers ranged from moderate to excellent, which was relatively higher in CT with DLR (*κ* = 0.69‒0.82) than CT with hybrid IR (*κ* = 0.57‒0.73).

**Table 3:** Comparisons of qualitative image analyses

	Hybrid IR		DLR		
		kappa value		kappa value	*p* value
Overall image quality	2.98 ± 0.58	0.57	4.09 ± 0.68	0.69	< 0.001
Image noise	2.61 ± 0.61	0.73	3.92 ± 0.74	0.82	< 0.001
Sharpness	3.17 ± 0.61	0.65	4.03 ± 0.64	0.72	< 0.001
Vessel conspicuity	3.56 ± 0.64	0.68	4.23 ± 0.61	0.76	< 0.001
Lesion delineation	3.78 ± 0.68	0.62	4.03 ± 0.69	0.70	< 0.001

Data are means ± standard deviations

*IR* iterative reconstruction, *DLR* deep learning reconstruction

**Fig. 5:**
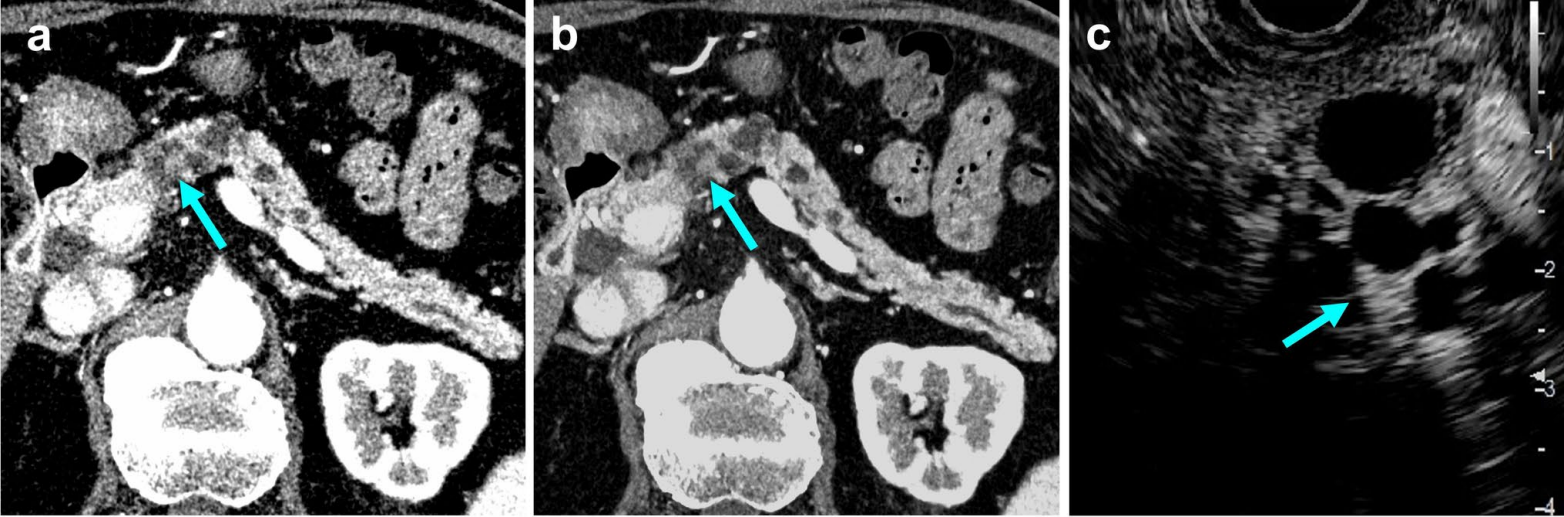
Pancreatic phase UHR-CT images in an 81-year-old man who had pathologically confirmed branch-duct type IPMN with high-grade dysplasia. **a** Image with hybrid IR shows a lobulated cystic lesion measuring 23-mm in diameter in the pancreas body. The lesion communicates with the MPD and enhanced thickened walls (arrow) are suspected with intermediate diagnostic confidence (score 3 and 4 for readers). **b** Image with DLR shows enhanced thickened walls (arrows) with higher diagnostic confidence (score 4 for both readers). **c** Contrast-enhanced ultrasound image shows enhanced thickened walls in a cystic lesion of the pancreas body (arrows). *UHR-CT* Ultra-high-resolution computed tomography, *IPMN* Intraductal papillary mucinous neoplasm, *IR* Iterative reconstruction, *MPD* Main pancreatic duct, *DLR* Deep learning reconstruction

**Fig. 6:**
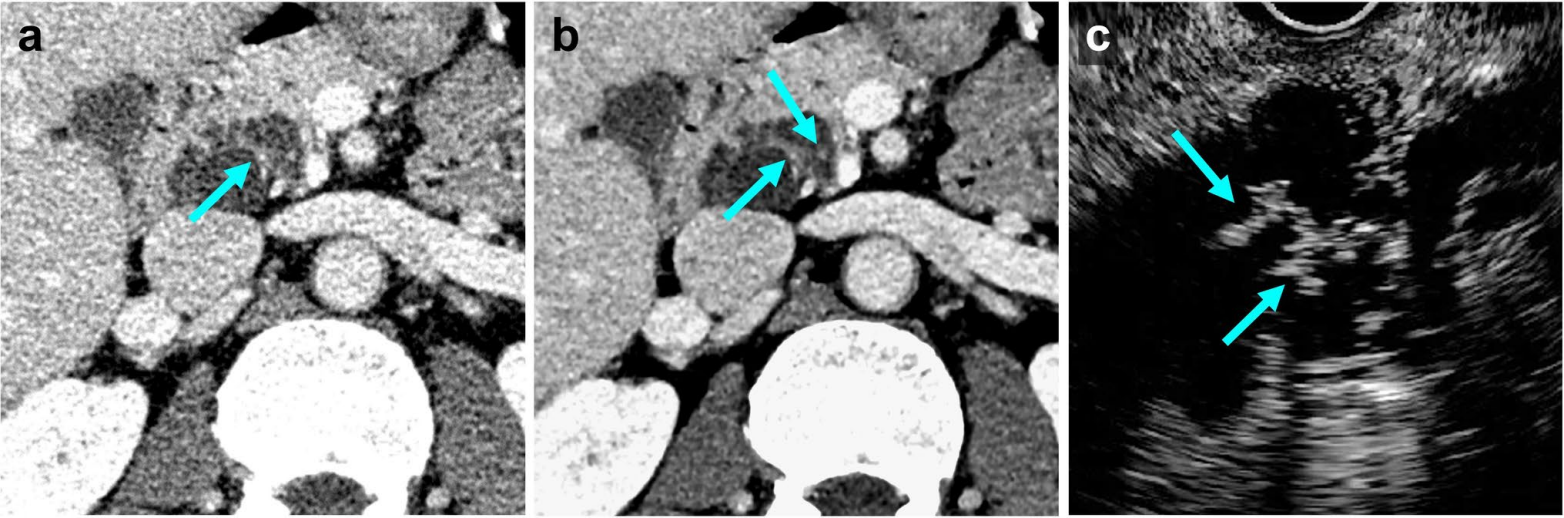
Portal venous phase UHR-CT images in a 72-year-old woman who had pathologically confirmed branch-duct type IPMN with high-grade dysplasia. **a** Image with hybrid IR shows a lobulated cystic lesion measuring 33 mm in diameter in the pancreas head. Enhanced thickened walls (arrow) are suspected with intermediate diagnostic confidence (score 3 for both readers). **b** Image with DLR clearly shows enhanced thickened walls and enhanced mural nodules < 5 mm (arrows) with higher diagnostic confidence (score 4 and 5 for readers). **c** Contrast-enhanced ultrasound image shows enhanced mural nodules in a cystic lesion of the pancreas head (arrows). *UHR-CT* ultra-high-resolution computed tomography, *IPMN* intraductal papillary mucinous neoplasm, *IR* iterative reconstruction, *DLR* deep learning reconstruction

The diagnostic performance of the imaging findings on CT with hybrid IR and DLR is summarized in Table 4. For evaluating continuity between the cystic lesion and the MPD, sensitivity did not show significant differences for CT with DLR (78.9%, 78.9%) and CT with hybrid IR (68.4%, 71.1%). Specificity was similar between CT with DLR (71.4%, 85.7%) and hybrid IR (71.4%, 71.4%) (*p* = 0.999 and 0.318). Overall accuracy was slightly higher for CT with DLR (77.8%, 80.0%) than CT with hybrid IR (68.9%, 71.1%) but did not reach significant differences (*p* = 0.157 and 0.189). Although the AUROC for CT with DLR (0.887, 0.927) was higher than that of CT with hybrid IR (0.827, 0.835), only reader 2 reached a significant difference (*p* = 0.028) but reader 1 did not (*p* = 0.072) (Fig. 5). For the assessment of enhanced thickened walls or enhanced mural nodules, sensitivity did not show significant differences for CT with DLR (58.3%, 58.3%) and CT with hybrid IR (41.7%, 50.0%) (*p* = 0.284 and 0.547). Specificity was similar between CT with DLR (93.9%, 97.0%) and hybrid IR (93.9%, 93.9%) (*p* = 0.999 and 0.712). Overall accuracy was slightly higher for CT with DLR (84.4%, 86.7%) than for CT with hybrid IR (80.0%, 82.2%) without significant differences (*p* = 0.142 and 0.128). The AUROC for CT with DLR (0.843, 0.894) was higher than CT with hybrid IR (0.785, 0.801), and reader 2 reached a significant difference (*p* = 0.040) but reader 1 did not (*p* = 0.063) (Fig. 6).

**Table 4:** Diagnostic ability for the pancreatic cystic neoplasms

	Hybrid IR		DLR		
		95% CI		95% CI	*p* value
Continuity between the cystic lesion and the main pancreatic duct					
Reader 1					
Sensitivity	68.4% (26/38)	62.3‒72.1%	78.9% (30/38)	72.9‒82.6%	0.248
Specificity	71.4% (5/7)	38.3‒91.4%	71.4% (5/7)	38.8‒91.3%	0.999
Accuracy	68.9% (31/45)	58.6‒75.1	77.8% (35/45)	67.6‒84.0%	0.157
AUROC	0.816	0.623‒0.989	0.887	0.751‒1.000	0.072
Reader 2					
Sensitivity	71.1% (27/38)	65.0‒74.7%	78.9% (30/38)	72.8‒81.1%	0.213
Specificity	71.4 (5/7)	38.4‒91.4%	85.7% (6/7)	52.4‒97.4%	0.318
Accuracy	71.1% (32/45)	60.8‒77.3%	80.0% (36/45)	69.6‒83.6%	0.189
AUROC	0.827	0.627‒0.991	0.938	0.842‒1.000	0.028
Enhanced thickened wall or enhanced mural nodule					
Reader 1					
Sensitivity	41.7% (5/12)	22.7‒53.2%	58.3% (7/12)	37.5‒69.8%	0.284
Specificity	93.9% (31/33)	87.1‒98.1%	93.9% (31/33)	86.4‒98.1%	0.999
Accuracy	80.0% (36/45)	69.9‒86.2%	84.4% (38/45)	73.3‒90.6%	0.142
AUROC	0.785	0.615‒0.956	0.843	0.698‒0.989	0.063
Reader 2					
Sensitivity	50.0% (6/12)	29.9‒61.5%	58.3% (7/12)	38.6‒65.1%	0.547
Specificity	93.9% (31/33)	86.6‒98.1%	97.0% (32/33)	89.8‒99.4%	0.712
Accuracy	82.2% (37/45)	71.5‒88.4%	86.7% (39/45)	76.1‒90.3%	0.128
AUROC	0.801	0.634‒0.967	0.916	0.834‒0.993	0.040

Data are expressed as frequency (count)

*IR* iterative reconstruction, *DLR* deep learning reconstruction, *95% CI* 95% confidence interval, *AUROC* area under the receiver operating characteristic curve

## Discussion

This study investigated the clinical utility of integrating DLR with UHR-CT for evaluating PCNs. By addressing the inherent limitations of UHR-CT in the abdomen, particularly the increased image noise due to smaller detector elements, the DLR algorithm improved both the objective image quality and diagnostic confidence in imaging findings associated with PCNs. Our findings indicate that the DLR algorithm is a promising solution for detecting and characterizing PCNs using UHR-CT.

One of the challenges of UHR-CT with higher spatial resolution that has thinner slices is the risk of higher image noise-degrading image quality compared to conventional CT [12–14]. The most significant advantage of DLR is its ability to effectively reduce image noise while preserving image texture and spatial resolution [5–7]. Our quantitative results showed that although CT values were slightly higher on the images with DLR than those with hybrid IR, DLR significantly reduced image noise and improved the CNR of abdominal structures compared to hybrid IR, aligning with findings from previous studies [14, 16, 17]. Qualitative assessments further demonstrated the advantages of DLR; the overall image quality, sharpness, and vessel conspicuity were consistently higher when using DLR on UHR-CT. Lesion delineation of PCNs was also significantly improved by applying DLR compared to hybrid IR. Previous studies have argued that image quality was better on DLR than hybrid IR and model-based IR abdominal UHR-CT images because DLR reduced low-frequency noise components [16, 21]. These advantages of DLR in abdominal CT could be particularly relevant for detecting the imaging features of PCNs, which are crucial for characterization and risk stratification [3, 4]. Furthermore, the interobserver agreement for qualitative assessments improved with DLR, as reflected by higher kappa values. This consistency underscores the potential of DLR to standardize diagnostic interpretations across readers, thus reducing inter-reader variability in clinical practice.

Our findings further highlight the clinical relevance of UHR-CT by demonstrating potential utility for PCNs. Continuity between the cystic lesion and main pancreatic duct is a critical feature for distinguishing IPMN from other PCNs [3, 4, 22]. Song et al. [23] reported that MRI was significantly more accurate than CT in predicting ductal communication in cystic lesions, in which the AUROC of CT was 0.774‒0.790 and that of MRI was 0.916‒0.949. In terms of intra-cystic structures, the presence and size of enhancing mural nodules have been reported as one of the strongest associations with malignancy risk in PCNs [24–28]. In this study, DLR produced a higher AUROC than hybrid IR for continuity between the cystic lesion and MPD (0.887‒0.938 vs. 0.816‒0.827) and enhanced thickened walls or enhanced mural nodules (0.843‒0.916 vs. 0.785‒0.801). Although DLR did not significantly improve sensitivity, specificity, or accuracy in our study, higher diagnostic confidence for the evaluation of imaging findings associated with PCNs produced a more reliable lesion characterization and patient management [3, 4]. The use of UHR-CT may improve the accuracy of management strategies and reduce the need for CE-EUS that is typically performed in high-risk patients but it is invasive, high-cost, and operator-dependent [5–7]. On the other hand, imaging findings associated with PCNs could not be detected even with the use of DLR in several cases maybe due to the intrinsic resolution limit of CT examination. Moreover, no direct comparison between UHR-CT and conventional resolution CT was performed in this study, and further studies are required to clarify the clinical advantages of UHR-CT over conventional resolution CT for the diagnosis of PCNs.

Increasing radiation exposure is a trade-off for high spatial resolution on UHR-CT, and radiation doses were relatively higher with reference to diagnostic reference levels [29]. While the potential increase in radiation exposure warrants careful consideration, it must be balanced against the clinical benefit of improved diagnostic capacity. Although the UHR-CT scanner can improve the detectability of small or subtle lesions [13, 16], maintaining diagnostic performance requires higher radiation doses compared with conventional CT scanner, even though lower dose examination could be achieved by DLR [17, 18]. Super-resolution DLR can simultaneously improve spatial resolution and reduce image noise, which may be useful even for the abdominal region [30]. In addition, the recently introduced photon-counting detector CT improves the overall geometric efficacy by eliminating the need for interpixel optical reflectors, enabling an in-plane spatial resolution of 0.125 mm without increasing radiation exposure [31–33]. Future studies should investigate whether these newly developed techniques improve the diagnostic performance for the diagnosis of PCNs in UHR-CT without increasing the radiation dose.

This study has several limitations. First, the single-institution retrospective design with a relatively small sample size has potential biases. Multicenter studies with larger numbers of patients are needed to validate the clinical utility of DLR in patients with PCNs. Second, histopathological confirmation was not obtained for any of the PCNs. Third, we acquired the UHR-CT images at a sufficient radiation dose. It may be possible to reduce the radiation dose while maintaining image quality when DLR is applied to abdominal CT [17]. Further investigations are required to verify the applicability of dose reduction in UHR-CT of the abdomen using the DLR algorithm. Fourth, different reconstruction strengths were selected between the hybrid IR and DLR, and this could have influenced the image quality comparisons in this study. Finally, we evaluated a single vendor-specific DLR algorithm. Further studies using other algorithms, including those from different vendors, are essential to establish generalizability and determine whether these benefits extend across platforms.

In conclusion, DLR improved image quality and diagnostic performance by effectively reducing image noise and improving lesion conspicuity in the diagnosis of PCNs on UHR-CT. In addition, DLR demonstrated greater diagnostic confidence for the assessment of imaging findings associated with PCNs. These findings support the implementation of DLR in routine clinical abdominal UHR-CT.

## Data Availability

The datasets used and/or analyzed during the current study are available from the corresponding author on reasonable request.

## Abbreviations

PCN: Pancreatic cystic neoplasm
IPMN: Intraductal papillary mucinous neoplasm
CE-EUS: Contrast-enhanced endoscopic ultrasound
MPD: Main pancreatic duct
UHR-CT: Ultra-high-resolution CT
DLR: Deep learning reconstruction
IR: Iterative reconstruction
PP: Pancreatic phase
PVP: Portal venous phase
CTDI_vol_: Volume CT dose index
DLP: Dose length product
HU: Hounsfield unit
ROI: Region of interest
CNR: Contrast-to-noise ratio
AUROC: Areas under the receiver operating characteristics curve
